# Colocolic Intussusception in an Older Child: A Rare Case Report and a Literature Review

**DOI:** 10.1155/2013/106831

**Published:** 2013-02-28

**Authors:** Anupam Das, Lalmalsawma Ralte, A. S. Chawla, S. V. Arya, Anil Kumar, Ravi Saroha, Dheer Singh Kalwaniya

**Affiliations:** Department of General Surgery, Vardhman Mahavir Medical College, Safdarjung Hospital, Ansari Nagar, New Delhi 110029, India

## Abstract

Intussusception is a common cause of intestinal obstruction and colicky abdominal pain in the children, particularly infants, the commonest being the ileocolic variety with colocolic variety being a very rare entity. We present a case of colocolic intussusception in a 13-year-old boy which is otherwise seen in adults, precipitated by colonic malignancies. The patient presented with acute abdominal pain and bleeding perrectum with obstipation for 7 days. Preoperative USG abdomen was normal, and abdominal X-rays showed multiple air fluid levels. Intraoperative findings included a polypoidal growth in the descending colon as the leading point with the formation of a colo-colic intussusception. Following reduction and segmental resection, histopathology reported mucinous adenocarcinoma of colon which is also a rarity in pediatric age group. This case has been compared with previous cases reported in the literature.

## 1. Introduction

Intussusception occurs when one portion of the gut becomes invaginated within an immediately adjacent segment, almost invariably, proximal into the distal. The condition is seen most commonly in the children with the peak incidence between 5 and 10 months of age. About 90% cases are idiopathic. The majority of cases occur in the region of the ileocecal valve, and no lead point can be precisely identified. Other types of intussusception that are rarer and have an anatomic lead point include ileoileal, colocolic, and ileoileocolic. Almost all cases of colocolic intussusception occur with a lead point such as polyp or tumoral mass. In a significant number of these cases, juvenile polyps were identified as leading point.

Intussusception lead points are more common in neonates, older children, and cases restricted to the small intestine. Colocolic intussusception in the adults is almost always a complication of preexisting colonic disease, usually carcinoma or polypoid tumor. Pediatric patients presenting with documented colocolic intussusception should suggest the possibility of a colonic polyp or other mass lesions.

## 2. Case Presentation

 A 13-year-old child was admitted in the emergency ward of the Department of General Surgery, Safdarjung Hospital, with complaints of abdominal pain for 15 days and repeated episodes of vomiting and passing blood in the faeces with abdominal distension for 7 days and obstipation for the previous day. The pain was initially colicky in nature more so on the left lower quadrant of the abdomen but at a later date progressed to being continuous. Initially the pain was accompanied by gradual distension of the abdomen followed by vomiting. There was history of passage of worms per rectum 1 month back associated with low-grade fever. There were no bladder complaints, jaundice, weight loss, similar episodes previously, tuberculosis, or exposure to tuberculosis. The patient had underwent left-sided orchidopexy for undescended testis at 3 years of age.

**Figure 1:**
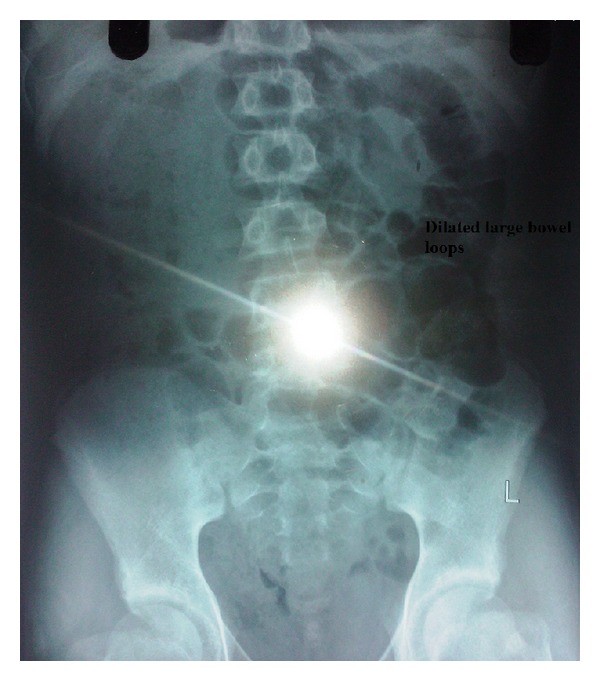
Erect abdominal X-ray showing dilated large bowel loops.

Physical examination showed distended abdomen and tenderness in the left iliac fossa. No separate lump was palpable. Digital rectal examination revealed red currant jelly stools, and anal canal was found to be ballooned. Bowel sounds were absent. The rest of the systemic examination was found to be unremarkable. Among the blood investigations, hemogram, kidney, and liver function tests, serum electrolytes were found to be within normal range. The erect abdominal X-rays revealed dilated large bowel loops signifying distal intestinal obstruction (Figure 1). The ultrasound of the abdomen showed mild splenomegaly (12.5 cm), 11 mm right renal calculus in the middle calyx with no hydronephrosis, and mild interbowel-free fluid with no signs suggestive of intussusception. Based on these preoperative clinicoradiological findings, a diagnosis of large bowel obstruction was made, and decision was taken to perform a laparotomy.

**Figure 2:**
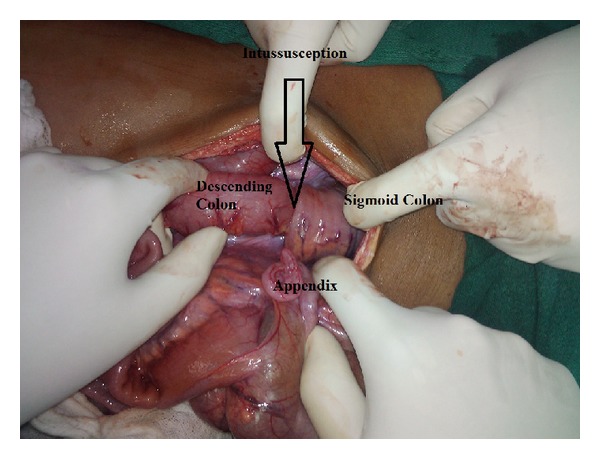
Photograph showing the colocolic intussusception.

The laparotomy was undertaken using the midline incision, and the following findings were noted:

1. small bowel collapsed, ileocecal junction was found to be normal;
2. descending colon-sigmoid colon intussusception with a 3 cm × 3 cm intraluminal polypoidal growth in the descending colon acting as the lead point, with the sigmoid colon as the intussuscipiens (Figure 2).

**Figure 3:**
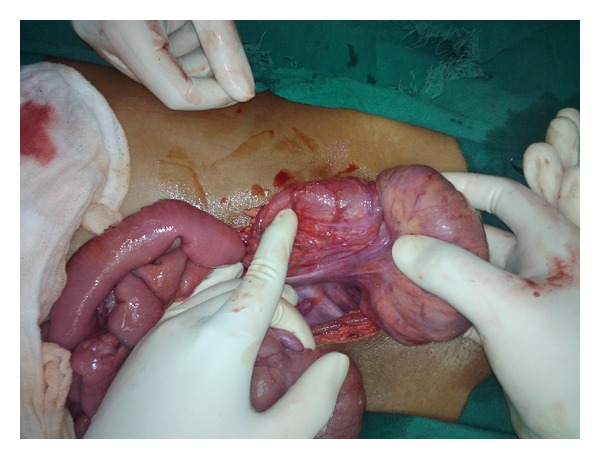
Intussusception being reduced intra-operatively.

**Figure 4:**
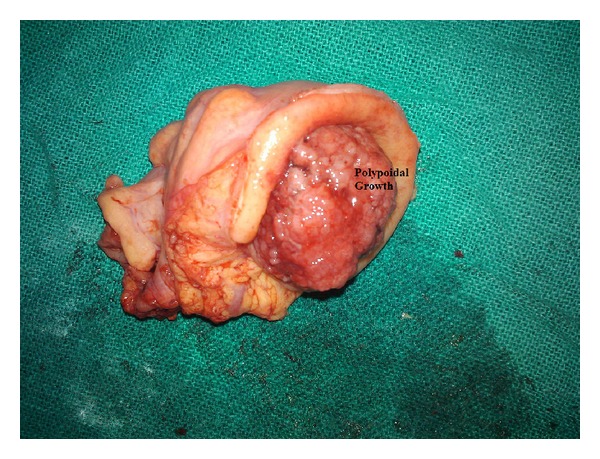
Resected specimen showing the polypoid growth with irregular luminal surface in the descending colon.

The intussusception was reduced manually (Figure 3), and segmental resection was done taking 5 cm margins on either side (Figure 4). Colo-colic anastomosis was performed in double layers, and a protective loop ileostomy was created 1 and 1/2 feet proximal to the ileo-cecal junction. The specimen was subjected to histopathological examination. 

Postoperatively the patient was allowed oral clear fluids on the first postoperative day and semisolid diet on the second postoperative day. The patient was discharged on the fourth postoperative day. 

The histopathological examination of the resected specimen diagnosed it to be mucinous adenocarcinoma of the colon with the resected margins microscopically free from the tumor. The patient in the followup period underwent contrast enhanced CT scan of the abdomen, pelvis and thorax, CEA levels, and colonoscopy to look for synchronous lesions, all were found to be normal, and the child was thereafter referred to the Medical Oncology and Radiation Oncology Department where he is being considered for adjuvant therapy and is under fortnightly follow-up.

## 3. Discussion

Adult colo-colic intussusception arises most commonly as a complication of preexisting colonic disease, usually carcinoma or polypoid tumor [1]. Pediatric patients with colocolic intussusception should suggest the possibility of a colonic polyp or mass lesion [1]. Although Mahmudloo et al. reported a case of colo-colic intussusception without a pathologic lead point in a 7-year-old boy [1], but the majority of the case reports in the literature reported juvenile polyps responsible for this variety of intussusception in the pediatric age group [2]. Similar cases caused by juvenile colonic polyp in pediatric age group were reported by Arthur et al. [3] and Abrahams et al. [4].

Pediatric colo-colic intussusception caused by malignancy has been reported rarely in the literature. Soccorso et al. reported a case of sigmoid colon ganglioneuroma in a 5-year-old child who presented with intermittent colo-colic intussusception [5]. Although the leading point in our case was grossly similar to that in other reported cases, the histopathology was different. The clinical presentation of the patient was however quite similar, that is, abdominal pain, vomiting, hematochezia, and intestinal obstruction.

Colon cancer is rare in younger age groups particularly without known risk factors. Colon cancer in young age is more likely to be diagnosed at advanced stage, to present unfavorable tumor histology such as mucinous adenocarcinoma and poor outcome. The review of the literature suggests that colon carcinoma in children and adolescents is predisposed by syndromes like Turner Syndrome or familial adenomatous polyposis. Chung et al. reported a case of mucinous adenocarcinoma in a 19-year-old patient who presented with abdominal distension and severe abdominal tenderness in the left lower quadrant [6]. The case which we report here is more similar to presentation of colonic carcinoma in adult age group although that is too rarely as reported by Dan et al. in a 27-year-old man diagnosed on CECT abdomen [7]. 

Colorectal carcinoma in children is very uncommon and could be easily misdiagnosed, resulting in advanced stage disease at diagnosis. Because radical surgery which is the mainstay of treatment is possible only in patients with early stage disease, a high level of awareness and early diagnosis are critical [8]. 

## References

[1] Mahmudloo R, Gheibi S, Vahed SN (2008). Colocolic intussusception without lead point; A case report and literatare review. *Iranian Journal of Pediatrics*.

[2] Ippolito RJ, Touloukian RJ (1978). Colocolic intussusception in an older child. Caused by a polyp of the distal colon. *Clinical Pediatrics*.

[3] Arthur AL, Garvey R, Vaness DG (1990). Colocolic intussusception in a three-year-old child caused by a colonic polyp. *Connecticut Medicine*.

[4] Abrahams RB, Franco A, Lewis KN (2012). Pediatric colocolic intussusception with pathologic lead point: a case report. *Journal of Medical Cases*.

[5] Soccorso G, Puls F, Richards C, Pringle H, Nour S (2009). A ganglioneuroma of the sigmoid colon presenting as leading point of intussusception in a child: a case report. *Journal of Pediatric Surgery*.

[6] Chung MY, Park YS, Ryu SR (2012). A case of colonic mucinous adenocarcinoma in 19-year-old male patient. *Clinical Endoscopy*.

[7] Dan JM, Agarwal S, Burke P, Mahoney EJ (2012). Adult intussusception secondary to colorectal cancer in a young man: a case report. *Journal of Emergency Medicine*.

[8] Salas-Valverde S, Lizano A, Gamboa Y (2009). Colon carcinoma in children and adolescents: prognostic factors and outcome-a review of 11 cases. *Pediatric Surgery International*.

